# Bang-Bang Control of Feeding: Role of Hypothalamic and Satiety Signals

**DOI:** 10.1371/journal.pcbi.0030097

**Published:** 2007-05-25

**Authors:** B. Silvano Zanutto, John E. R Staddon

**Affiliations:** 1 Instituto de Ingeniería Biomédica–Universidad de Buenos Aires, Buenos Aires, Argentina; 2 Instituto de Biología y Medicina Experimental–Consejo Nacional de Investigaciones Científicas y Técnicas, Buenos Aires, Argentina; 3 Department of Psychology and Neuroscience, Duke University, Durham, North Carolina, United States of America; University College London, United Kingdom

## Abstract

Rats, people, and many other omnivores eat in meals rather than continuously. We show by experimental test that eating in meals is regulated by a simple bang-bang control system, an idea foreshadowed by Le Magnen and many others, shown by us to account for a wide range of behavioral data, but never explicitly tested or tied to neurophysiological facts. The hypothesis is simply that the tendency to eat rises with time at a rate determined by satiety signals. When these signals fall below a set point, eating begins, in on–off fashion. The delayed sequelae of eating increment the satiety signals, which eventually turn eating off. Thus, under free conditions, the organism eats in bouts separated by noneating activities. We report an experiment with rats to test novel predictions about meal patterns that are not explained by existing homeostatic approaches. Access to food was systematically but unpredictably interrupted just as the animal tried to start a new meal. A simple bang-bang model fits the resulting meal-pattern data well, and its elements can be identified with neurophysiological processes. Hypothalamic inputs can provide the set point for longer-term regulation carried out by a comparator in the hindbrain. Delayed gustatory and gastrointestinal aftereffects of eating act via the nucleus of the solitary tract and other hindbrain regions as neural feedback governing short-term regulation. In this way, the model forges real links between a functioning feedback mechanism, neuro–hormonal data, and both short-term (meals) and long-term (eating-rate regulation) behavioral data.

## Author Summary

Why do rats, people, and many other animals eat in bouts (meals) rather than continuously? How are they able to regulate not only total intake over a long period, but also within a meal? Is there a simple physiological explanation for these facts? We introduce a feedback model for eating regulation that can be readily interpreted in terms of existing neural data. We show by experiment and a theoretical model that eating in meals is regulated by a simple control system, like a house thermostat. In an experiment, we studied the meal patterns in rats after an interruption in food availability just as the animal tried to start a new meal. The results of this type of experiment cannot be explained by existing homeostatic approaches. We propose a simple model that combines long-term regulation (over months and years) by signals that reflect metabolic conditions, providing the set point for negative feedback, and short-term regulation (within a meal or a day) by feedback from the delayed gastrointestinal aftereffects of eating. The model shows that the dynamic process that governs regulation, meal patterns, and even adaptation to some complex operant food-reinforcement schedules is remarkably simple: ingested feeding inhibits eating, but the inhibitory effect is delayed.

## Introduction

Feeding, the prototypical motivational system, involves both internal and external factors; it can be studied both behaviorally by looking at patterns of eating, and physiologically by looking at neural and hormonal factors that initiate or suppress eating. Historically, most attention has been devoted to internal factors, such as levels of circulating glucose and lipid-related hormones [1–3]. Eating rate is affected by external factors such as taste (the evolutionary predictor of food quality), learning and habits, social situation, stress and emotion, and many others, and the way that these variables interact is complex and not fully understood [4,5]. Nevertheless, there are some simple regularities in temporal patterns of eating. Most omnivores, such as rats and guinea pigs—as well as human beings—eat not at fixed intervals of time, or at random times, but in *meals,* bouts of concentrated eating that are approximately periodic and separated by periods when behavior other than eating occurs. The duration and frequency of meals adjust to perturbations such as food deprivation or forced feeding in such a way as to regulate overall food intake [6–8]. Common observation tells us that in the presence of ad libitum food, eating probability increases with time, but then once eating has begun, its probability decreases with time [9]. The dynamics look relatively simple—simple enough that almost no research has been done on the dynamics of eating per se and still less on the possible role of neural mechanisms in behavioral dynamics.

The first step is to find the neuropsychological components that correspond to parts of the control circuit for feeding (comparator, feedback loop, and reference). Endogenous factors that modify the size of an ongoing meal are called satiety signals (SSs). These signals, generated during and after a meal, provide information to the brain that inhibits feeding and leads to meal termination [10]. The SSs are generated in the gastrointestinal tract and abdominal viscera, as well as in the oral cavity (i.e., taste factors [11,12]). They provide information about mechanical (e.g., stomach stretch, volume) and chemical properties of food (e.g., via peptides such as cholecystokinin, ghrelin, and peptide YY [PYY]), which have been linked to short-term (within-a-day) feeding behaviours [13]. Other peptides secreted from the gastrointestinal system have been reported to control meal size when administered systemically [14–17]. In addition, amylin [18] and glucagons [19], which are secreted from the pancreatic islets during meals, also reduce meal size. SSs are relayed to the hindbrain, mainly to the nucleus of the solitary tract (NTS), either indirectly via nerves from the gastrointestinal tract, especially the vagus (e.g., cholecystokinin and glucagon), or else circulate via the blood and interact with local receptors in the hindbrain (e.g., amylin) [20].

The hindbrain (mainly the NTS) also receives, via several hypothalamic nuclei, signals that reflect the fat mass of the body. The best-known signals are the adiposity signals leptin and insulin—hormones secreted into the blood in direct proportion to the amount of stored body fat. Leptin is secreted from fat cells (adipocytes) in direct proportion to the amount of stored fat [21,22]. Insulin is secreted from pancreatic β cells in response to increases of glucose. Moreover, basal insulin in the absence of elevated glucose, as well as every increment of insulin above baseline during meals, is in direct proportion to total body fat or adiposity [23,24]. Obese individuals have relatively high basal insulin, whereas lean individuals have relatively low levels [25]. In this way, circulating leptin and insulin levels are each a good indicator of body fat, and both hormones are able to enter the brain from the blood and stimulate specific neural receptors.

These two adiposity signals (leptin especially) have been linked to longer-term weight regulation (over months and years) [13]. Some gut-related peptides are also long-term regulators. Rodents and humans with reduced PYY levels in response to food intake tend toward obesity, for example. Chronic administration of PYY reduces adiposity in rodents [26]. Also, PYY-null mice (unable to produce the hormone because the gene for PYY has been knocked out) are hyperphagic and develop marked obesity but are hypersensitive to exogenous PYY. Moreover, chronic treatment with PYY reverses their obesity phenotype [27]. The effect of this hormone was also studied in obese children, where there is a reciprocal relationship between obesity and PYY [28].

Finally, animals and humans with defects in the central melanocortin system display a characteristic melanocortin obesity phenotype characterized by increased adiposity and hyperphagia [29]. The central melanocortin system interacts with long-term regulators of energy homeostasis such as leptin and also with the gut-released peptides involved in the short-term regulators (e.g., cholecystokinin, ghrelin, and PYY). All these data suggest that there is a large degree of redundancy in the orexigenic (appetite-stimulating) pathways, showing an evolutionary bias toward energy storage. Despite this redundancy, the neurophysiological pathways suggest that feeding is regulated by a feedback loop, where the hypothalamus provides the long-term regulatory input to the NTS that acts as the set point. It also receives SSs as feedback inputs, acting as short-term regulators. The SSs have been referred to as direct controls [30], because food acts directly on receptors along the gastrointestinal tract. All other controls, such as metabolic, rhythmic, and ecologic, have been referred to as indirect controls [30]. They act by modulating the central effects of the direct controls.

Many areas of the brain are sensitive to long-term regulators. Leptin receptors have been found on paraventricular nucleus (PVN) and lateral hypothalamic (LHA) neurons, implicating them as direct targets for regulation by circulating adiposity signals. PVN stimulation inhibits food intake, whereas the opposite is true of stimulation of the LHA [31] and adjacent perifornical area [32]. Conversely, bilateral PVN lesions cause a hyperphagic obesity syndrome, whereas bilateral lesioning of the LHA causes anorexia and weight loss [31,33]. Consistent with these results, several neuropeptides synthesized in PVN neurons reduce food intake and body weight when administered centrally. Hypothalamic areas including the PVN, zona incerta, perifornical area, and LHA are richly supplied by axons from the arcuate nucleus, which has greater concentrations of leptin and insulin receptors than other hypothalamic sites [34–38]. The arcuate nucleus has at least two distinct populations of neurons with opposing actions on food intake, responding not only to leptin and insulin, but also to gut hormones (the best studied are ghrelin and, recently, PYY). The first population produces orexigenic neuropeptide Y and agouti-related protein (NPY/AgRP). The second population produces the anorexigenic (appetite-suppressing) proopiomelanocortin and cocaine and amphetamine–regulated transcript (POMC/CART) [17,39–41].

The NTS has been identified as a “satiety center” [13]. Several workers have suggested that the NTS integrates inputs transmitted through the vagus and sympathetic fibers [42–44] and hypothalamic input [34,45–48] involved in energy homeostasis [34]. When it is lesioned in rats, it causes them to eat less and even starve to death [13,49]. In this way, net neuronal output from the NTS (and other hindbrain regions) controls meal size [50]. Taken together, these facts about neural targets in the NTS provide the ingredients for a simple homeostatic account of the nonassociative (i.e., unlearned) aspects of feeding dynamics.

Even though it is generally accepted that depletion of the body's energy reserves can cause eating at a time when it would not normally occur, current thinking is that most meals are initiated at times that are convenient or habitual—based on social or learned factors rather than on the regulation of energy balance. Nevertheless, animals continue to regulate their food intake even under constant environmental conditions, implying the existence of some basic regulatory process, albeit one that is normally overlaid by the effects of learning. If the competition among motivational systems (hunger versus sex, versus thirst, etc.) is ignored for the moment, the endogenous factor is regulatory and tends to oppose anything that forces eating rate to be reduced below an optimal value, the set point. The exogenous factors are approximately additive and cause eating rates to be higher or lower than the set point; accordingly, they are positive or negative [5].

### An Integrative Model

A model for feeding dynamics gains support in several ways. It can explain existing data from behavioral studies in which food access is restricted in various ways: it can be tested experimentally via biochemical and neural interventions, and also by behavioral experiments explicitly designed to test model predictions. We have already shown that a simple lagged-effect (cascaded-integrator [CINT]), bang-bang control behavioral model can account for a wide range of existing behavioral data [51,52]. The CINT model provides a unifying account that can explain both eating-rate regulation and the broad features of meal duration and timing. It explains why rats adapt to changes in reward size by adjusting meal size rather than intermeal interval (IMI), and why interruption of feeding affects primarily the first postinterruption meal (PIM). It also explains the effects on eating rate of imposing a minimum interpellet interval as well as other operant-schedule constraints such as cost and “procurement size” in a wide variety of closed-economy experiments (see [53] and other papers in that issue for a review of these procedures). The bang-bang CINT model also accounts quantitatively for the complexities of meal–intermeal correlations [50] (see also [9,54]).

In previous work, we attempted to explain existing behavioral data in a unified way, but did not explicitly test the CINT model. We now show how the model can handle experimental data not explained by existing homeostatic feeding models. If the behavioral model can be readily interpreted in terms of existing neural data, the next step is to see how physiological manipulations affect its components.

We studied meal patterns in free feeding and after interrupting food access (for different amounts of time) at the moment when rats start a new meal. Meal duration and the time between meals (IMI) were compared under free-food conditions and after interruptions. The overall temporal patterns of eating, as well as correlations between preceding and following IMI and meal duration under these two conditions, were then compared with the predictions of the CINT model. With only a slight modification (to incorporate a limit on IMI duration), the same CINT model also fits real-time experimental data.

## Results

### Eating Pattern: Effect of Interruptions

**Figure 1:**
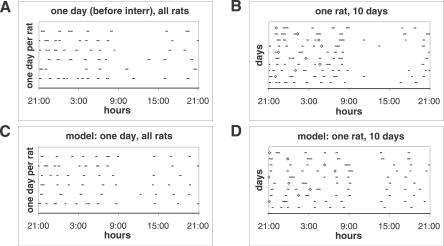
Meal Patterns in Rats under Free Feeding and with Unpredictable Interruptions Compares rasters of meals in a single rat and a group (A,B) and as simulated by the model (C,D). (A,C) Show the meals the day before beginning the interruptions for all rats (1 d per rat). (B,D) Shows ten consecutive days for a single rat, the first with no interruptions and the following with interruptions. The diamonds show times when the rat attempted to start a new meal, but was interrupted.

The eating pattern is shown in the raster plots of Figure 1. The top left panel shows meals the day before the beginning of interruptions for all rats, 1 d per rat. On the top right, there are ten consecutive days for an individual rat; the first day has no interruptions and the following days have them at onset times indicated by diamonds. The figure also shows that the model (bottom two panels), discussed in detail below, duplicates the general eating pattern.

**Figure 2:**
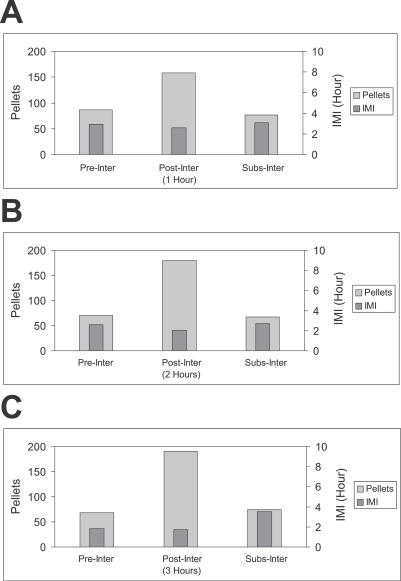
Effect of Interruptions on Meal Pattern Mean values of the number of pellets (clear gray columns) and the previous IMI (without interruption; thin dark gray columns) in the postinterruption (middle column), preceding (left column), and following (right column) meals. Three conditions are shown: when the interruption duration was 1 h (A), 2 h (B), or 3 h (C). In all cases, the number of pellets eaten in the first meal after the interruption (middle columns) is greater than in the preceding and subsequent meals (flanking columns), independent of the previous IMI.

The average size of meals (number of pellets eaten; *M*) preceding, just after, and after that (i.e., meal sizes M*_N−_*_1_ *,* M*_N_*, and M*_N+_*_1_, where meal *N* immediately follows an interruption) are shown in Figure 2. The data are analyzed for three interruption durations: 1, 2, or 3 h. The size of PIM size (middle light gray columns) is substantially larger than the size of preceding and subsequent meals (flanking light gray columns). The IMI preceding the meal (excluding the interruption period) is shown in the narrow dark gray columns: there is no relation between preinterruption interval and the PIM size.

**Figure 3:**
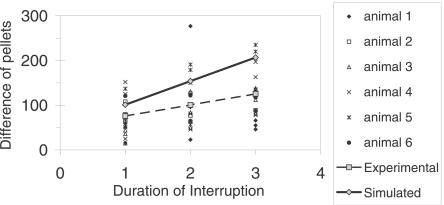
PIM Size as a Function of Interruption Duration Shows the difference between the number of pellets of the first meal after an interruption and the number of pellets of the previous meal as a function of duration of the interruption for six animals. The dashed line shows the linear regression of the experimental data. The solid line shows the linear regression of the simulated data.

A linear-regression analysis showed that the difference *y* between size of the first meal after the interruption (*N*) and preinterruption (*N* − 1) meal size (i.e., M*_N_* − M*_N−_*_1_), increased linearly as function of the interruption duration *x* (*F*_(1,52)_ = 7.86, *n* = 54, *p* = 0.007, *r^2^* = 0.13; *y* = 24.83*x* + 51.03). Thus, the number of pellets in the first meal after an interruption increases as a linear function of the interruption time, and is greater than the preceding and subsequent meals (Figure 3). Even though after the same interruption duration, not all meals have the same sizes (number of pellets), they are always relatively larger.

The size of the meal after the PIM was compared by one-way ANOVA with the sizes of other meals (excluding the PIM). The test shows no significant differences (*F*_(1,205)_ = 1.946, *p* > 0.1). Thus, an interruption affects *only the first PIM.* We have called this the *first-meal effect.*

To assess any pattern of IMIs under free-feeding conditions, IMIs after PIMs were compared with other IMIs at night (meals that began or ended in the daytime, and the PIM and following IMI, were excluded). The mean value of the first postinterruption IMI is 2.078 h, and the mean value of the second IMI is 1.43 h. One-way ANOVA shows a significant difference (*F*_(1,252)_ = 18.03, *p* < 0.0001). Thus, the IMI after the extra-large PIM is longer than usual. Nevertheless, there was no correlation between the sizes of individual PIMs and subsequent IMIs; and, as we said, the following meal size is not significantly different from the size of meals not perturbed by interruptions. In this way, the IMI after the PIM is longer than others, and the effect of interruption is compensated for *solely* by the PIM.

We also looked at the relationship between IMI and meal size for free-feeding meals that began and ended at night (as before). We wanted to see if, under free conditions, larger meals were followed by longer IMIs and vice versa. The answer was yes. Under homogeneous conditions in the “night” portion of the day–night cycle, IMI and meal size increased linearly as a function of previous meal size or IMI, respectively. Linear regression analysis showed that the IMI *y* increased as a function of the previous meal size *x* (*F*_(1,208)_ = 30.532, *n* = 210, *p* < 0.0001, *r^2^* = 0.1279; *y* = 0.01*x* + 0.683). Also, a linear-regression analysis showed the meal size *y* increased as a function of the previous IMI *x* (*F*_(1,207)_ = 10, *n* = 209, *p* < 0.005, *r^2^* = 0.046; *y* = 5.314*x* + 55.44).

**Figure 4:**
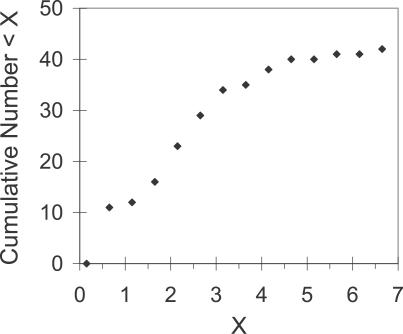
Cumulative Distribution of IMI PIM Sizes The values are calculated in bins of .5 h from .15 h to 6.15 h. The IMIs were measured at night; those that began or ended in daytime, and the PIM and the following IMI, were excluded. Less than 10% of the IMIs following PIMs are longer than 4.15 h.

Figure 4 shows the cumulative number of IMIs less than a given value. Less than 10% of IMIs are longer than 4.15 h, which seems to be an asymptote. For each animal, IMI values greater than 75% of its maximum value are reached after its previous PIMs of 97 pellets (the minimum size is 52 pellets and the maximum is 220 pellets). Even though there is no correlation between the PIM size and the following IMI, the fact that the maximum values are reached near the first quartile of the meal-size distribution shows that the postinterruption IMI has a saturation value.

**Figure 5:**
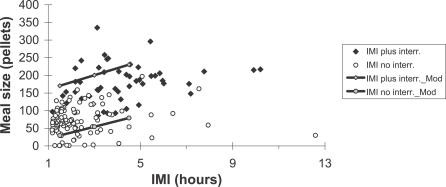
Effect on the Meal Sizes when Previous IMIs of Similar Length Include or Do Not Include Enforced Interruptions Compares the number of pellets eaten after IMIs with interruptions (filled diamonds) and others (measured in the same way as in Figure 4) without them (empty circles). Solid line shows the linear regression of the simulated data: the IMIs with interruptions (gray diamonds) and without (gray circles).

PIM sizes were compared with other meals after intermeal intervals of a similar length (of interruption plus the previous IMI) that occurred spontaneously (i.e., without interruptions). The IMIs with no interruption were chosen at night, as before. In the absence of interruption, the mean meal size was 71.32 pellets, and 176.12 pellets with interruption. The data were compared using one-way ANOVA, which showed significant differences (*F*_(1,154)_ = 185.26, *p* < 0.0001). Thus, interruption (plus the previous IMI) evokes a larger subsequent meal size than a spontaneous IMI of similar length (Figure 5).

### CINT Model of Feeding Regulation

The feedback loop is closed not solely by glucose, as in the glucostatic theory, but by SSs acting as short-term regulators. As we noted earlier, the NTS (and other hindbrain regions) integrates inputs transmitted through the parasympathetic and sympathetic fibers and blood as well as the hypothalamic input that provides the set point for longer-term regulation. Thus, the NTS output controls meal size, and may act as a comparator in a feedback loop in the CINT model [51,52].

The CINT model has three properties: (1) the SSs are simulated by one variable, a lagged aftereffect of eating; (2) feeding occurs when the SS declines below a *set point* (θ); and (3) when the SS falls below the set point, it turns on feeding in an all-or-none fashion (*bang-bang* control: the all-or-none assumption may need to be relaxed to take account of incentive effects; see [52], Chapter 9). We suggest that the set point corresponds to hypothalamic input to the NTS.

The value of the set point expresses the long-term motivation for eating. A low set point corresponds to high energy reserves, a high set point to low reserves.

The delay between eating and a rise in the SS is simulated by a cascade of leaky integrators: a first-order linear system is the simplest way to model a lagged effect.

### Simulations

The parameters of the model (see Methods) were chosen as follows: *a_1_* = λ, *a_i_* = *a_i−_*_1_ + λ(1 − *a_i−_*_1_), *b_i_* = 1.7(1- *a_i_*) (i.e., three stages, but only two free parameters). For all the simulations, the parameters were λ = 0.985, Φ = 1.1. One time step = 1 s. Because rats eat much more in the 12-h dark phase of these experiments than in the light phase, we allowed the set point, θ, to vary smoothly between two saturation values, θ_dark_ and θ_light_: θ (t + 1) = θ (t) × (1 − 0.00005) + 0.00005 × (θ_day_ + *noise*); θ_day_ could take two values depending on the simulated period (light or dark): θ_dark_ = 0.13, θ_light_ = 0.12.

To simulate observed spontaneous variation in meal size and IMI, the set point varied randomly by adding *noise =* white noise (amplitude = 0.007).

The set time to eat a pellet was 6 s, followed by a refractory period of 4 s before another pellet could be eaten. A meal began with the first pellet after *V_I_* < θ and was considered to end after 3 min of no eating (*V_I_* ≥ θ; the 3 min were not included in meal duration). At the beginning of each condition, *V_1_* and *V_2_* were set to 0; *V_3_* = .12, to allow for eating on the previous day. To simulate the limit on IMI, *V_I_* was bounded between zero and 0.0017 + θ.

This version of the CINT model matches the new data and better fits data simulated previously [51,52], for which we added the following assumption to translate eating tendency into operant lever pressing: when eating tendency >0, lever-press rate was 2/s (estimated from observational data). The simulation matched essentially all the statistical properties of free and interrupted eating just described. (1) The CINT model reproduces the eating pattern shown in the raster of Figure 1. On the bottom left there are simulated meals in the day before of the beginning of the interruptions for all rats: 1 d per rat is shown. On the bottom right, there are ten consecutive days of the same rat; the first day has no interruptions, and the following days have them, as in the actual experiment. (2) It explains (as in the previous version) the linear relations between IMI and meal size. (3) It immediately accounts for the “first-meal effect”: when eating is interrupted for a few hours at preprogrammed times, the first (and only the first) meal is extra long. Eating rate and meal size thereafter both revert to normal values [7,55]. The model also accounts for the data shown in Figure 2: even though our procedure is different from Le Magnen's [7], the effect is similar. (4) The model also explains how the difference between the first meal size (pellets) after the interruption and the pre-interruption meal increases linearly as a function of interruption duration. Because the SS value is bounded, the simulation fits the experimental data of Figure 3 (the solid line shows the simulated data and the dashed line the linear regression of data in rats). (5) Here, the effect of the interruption is compensated only in the first PIM. The larger PIM provokes larger subsequent IMI, but because there is a maximum IMI, the following meal size is not greater than average. (6) We compared the effect on meal size of an interruption plus its previous IMI with the effect of a spontaneous IMI of similar total duration. The easiest way to do this was to reduce θ after an arbitrary meal to get an IMI duration of interruption plus it previous IMI. Specifically, the white noise in the computation of θ has an amplitude of 0.05. In Figure 5, the solid line shows the simulated data: the IMIs with interruptions (gray diamonds) and without them (gray circles). The simulation fits the experimental data: the interruption plus the previous IMI provokes larger meal size than after an IMI of a similar length without interruptions.

## Discussion

For many years, perhaps beginning with Le Magnen [55], repletion–depletion has been implicitly assumed to be the process that underlies feeding behavior. This approach has led to numerous attempts to identify the physiological signal that triggers eating [2,9]. However, there was still some uncertainty about other parts of the physiological feedback loop.

Part of the problem may be that the black-box dynamics of the process are still not fully understood. Thus, our approach had been predominantly behavioral until the present article, which draws attention to recent physiological developments that bring the behavioral and physiological data into closer registry. As we said above, the NTS (and other hindbrain regions) integrates inputs transmitted through the parasympathetic and sympathetic fibers and blood: SSs controlling short-term regulation (over the day); and hypothalamic input controlling the long-term regulation (over months and years), mediating energy homeostasis. Thus, the NTS may act as a comparator, and the feedback loop is closed not by glucose, as in the old theory, but by short-term regulators: SSs. The long-term regulators act as the set point. The CINT model can explain these data. Here, we refine it to fit better the real-time experimental pattern of previous data and to simulate behavioral experiments designed as an explicit test and not explicable by existing feedback models.

First, we analysed the correlation between the meal and the following IMI, because there are contradictory results. Next, we found that at night under free conditions, larger meals were followed by longer IMIs, and vice versa. We studied the eating pattern in rats when feeding is unpredictably interrupted: we looked at meal patterns after an interruption initiated by an attempt by the animal to start a new meal. We found that the PIM is larger than the size of preceding and subsequent meals, and the difference between the number of pellets of the PIM and the number of pellets of the previous meal is a linear function of duration of the interruption. We also analysed the IMI after the PIM, and found that it is longer than usual, though smaller than the saturation value, and that there is no correlation between the two. The size of the meals after the PIM is not significantly different from other meals. These results show that IMI is not a linear function of the previous PIM, and there is a saturation value. Finally, the interruption (plus the previous IMI) evokes a larger subsequent meal size than a spontaneous IMI of similar length, indicating a change in the animal's motivation to eat.

In this CINT version, as in the previous one, the set point (long-term regulator) is slightly modulated by the light–dark cycle, but here, the transition between the two values is smooth, following an exponential function. The SSs (short-term regulators) are a delayed effect of eating—simulated as a cascade of integrators, the simplest way to simulate a delay. The effect of the saturation of the IMI following the PIM was simulated by truncating the SSs at a maximum value (independent of the size of the PIM). The SSs are feedback to the comparator, the output of which controls when to eat.

The bang-bang CINT model can explain many relevant experimental data and can easily incorporate new data. It is both regulatory (i.e., homeostatic—a physiological property) and generates feeding in meals (an adaptation to ecological considerations). It is also important to note that the major predictions of the CINT model—first-meal effect, meal–intermeal correlations, regulation of eating rate, effect of meal size on meal duration but not IMI, etc.—depend on the structure of the model, not on particular parameter values. We also note that this simple model does not deal with the kinds of interaction among motivational systems that are necessary to explain prandial drinking and other apparent deviations from homeostasis. The model does not attempt to explain learning and incentive effects (we have made some suggestions on this point in [52], Chapter 9).

The simulations make three simple points: (1) feeding regulation in rats is bang-bang rather than proportional control. Eating is regulated in on–off fashion under most conditions rather than being proportional to the difference between set point and SSs. (2) The satiating effects of eating are delayed in a way that can be modelled by a simple first-order linear system. Finally, (3) long-term regulation (i.e., control of body weight) is separable from short-term regulation (i.e., control of meal pattern). Long-term regulation is controlled by a set point provided by the hypothalamic input; short-term regulation is controlled by SSs that vary from minute to minute rather than over days.

## Materials and Methods

### Subjects.

The subjects were six experimentally naive 45-d-old male Sprague-Dawley rats.

### Apparatus.

An experimental chamber was located in a soundproof box maintained at 22 ± 2 °C with lights on from 9 a.m. to 9 p.m. Each stainless steel cage measured 43 cm wide × 31 cm deep × 21 cm high and was equipped with a drinking tube, a food cup with a 45-mg pellet dispenser, a running wheel, and a nest. The nest was made of black Plexiglas (19 cm wide × 14 cm deep × 11 cm high). Infrared photobeams monitored head entries into the food cup, pellet entry into the V-shaped cup, and the presence of the pellet in a cup. A clear red light and the noise of the pellet dropping signaled pellet-in-cup.

### Procedure.

Pellet delivery was scheduled as follows. When the rat's head broke the photobeam inside the feeder, the first pellet was delivered. Once the rat removed it, a new pellet was delivered. This process continued until a pellet remained uneaten for 10 min, when it was removed by an air puff. Water was freely available from a drinking tube mounted on one side of the cage.

The times when each pellet is dropped and eaten, the times each session was started and ended, the interruption, and duration times were all recorded to the nearest millisecond.

The rats were housed individually and continuously in the experimental chamber, except for a maintenance period of about 30 min each day, when they were weighed, food and water was replenished, and the apparatus was cleaned and tested (this is known as a “closed economy”). For the first 3 d of the experiment, each rat had free access to food to habituate it to the apparatus.

After the third day, food delivery was interrupted during the night (from 9 p.m. to 9 a.m.) at one of three randomly selected times and for one of three randomly selected durations so the animals could not anticipate either the onset or offset of food availability. The interruption duration was either 1, 2, or 3 h. There was only one interruption per day. The interruption period began when the animal put its head inside the feeder to start a new meal after one of three times: 10 p.m., 1 a.m., and 4 a.m., called the interruption time. Note that the interruption time specifies the time of food-availability onset in the same way as a response-initiated delay reinforcement schedule (i.e., interruption onset time is equal to the interruption time plus the time to the next response). For example, suppose that on a given night, the selected interruption time is at 4 a.m. for an interruption duration of 2 h. If the animal first looks for a pellet (interrupts the photobeam) at 4:45 a.m. (the interruption onset time), the interruption timer starts and runs for 2 h, at which point a pellet is delivered and the red signal light is turned on.

The interruption and duration times were chosen at random without replacement, one pair of times per night. In this way, in 9 d each animal was tested on all combinations.

The dependent variables in the experiment were M, defined as the number of pellets eaten in a meal, and IMI. The end of the meal is when a pellet is eaten and then followed by at least 3 min with no further eating. Meal onset is defined as the time when at least 3 min of no eating precedes the eating of a pellet. IMI is the time between meal end and meal onset.

### Model.

**Figure 6:**
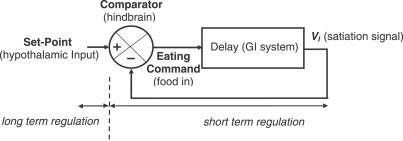
Sketch of the Model The block diagram of a system modeling long- and short-term regulation of feeding. Eating leads to delayed SSs (grouped in *V_I_*). When the SSs falls below the set point, the command is “eat”: otherwise, the command is “do not eat.”

To explain the new data, the CINT model was modified slightly (Figure 6) to include the observed limit on IMI values (Figure 4 and the fact that the maximum values are reached near the first quartile of the meal-size distribution) by imposing a similar bound on the SS.

If the feeding schedule is ad libitum (i.e., food is always available), then the equations for the I-unit cascaded system are:

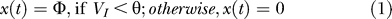
where x(*t*) is the satiation value which is input at each discrete time step, and Φ is a constant which is some function of the physical properties of the food (e.g., weight, type, caloric value, etc.) provided by the experimenter (i.e., bang-bang control): if the SS is below set point: eat (satiation value = Φ); otherwise, do not eat.

The SS is the output of a cascaded series of leaky integrators where the output of integrator *i* is the input to the next integrator *i* + 1. V_I_, the SS, is the output of the last integrator in the series, which is determined as follows. In discrete time, for the first integrator:

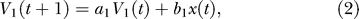
and for subsequent integrators:

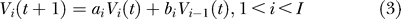
where *V_i_* is the state of integrator *i, a_i_* is the time parameter of integrator *i* (0 < *a_i_* < 1), and *b_i_* is the input weight. Two integrators are the minimum necessary to produce a delayed SS, but three (*I* = 3) gives a better fit to our data.

Eating occurs when *V_3_*, the output of the third integrator, falls below θ, and ceases when it exceeds θ.

## Abbreviations

CINT: cascaded integrator
IMI: intermeal interval
LHA: lateral hypothalamic
M: meal size
NTS: nucleus of the solitary tract
PIM: postinterruption meal
PVN: paraventricular nucleus
PYY: peptide YY
SS: satiety signal

## References

[pcbi-0030097-b001] Louis-Sylvestre J, Le Magnen J (1980). A fall in blood glucose level precedes meal onset in free feeding rats. Neurosci Biobehav Rev.

[pcbi-0030097-b002] Campfield LA, Smith FJ (2003). Blood glucose dynamics and control of meal initiation: A pattern detection and recognition theory. Physiol Rev.

[pcbi-0030097-b003] Mayer J, Thomas DW (1967). Regulation of food intake and obesity. Science.

[pcbi-0030097-b004] Weingarten HP (1983). Conditioned cues elicit feeding in sated rats: A role for learning in meal initiation. Science.

[pcbi-0030097-b005] Staddon JER (2003). Adaptive behavior and learning.

[pcbi-0030097-b006] Bare JK, Cicala G (1960). Deprivation and time of testing as determinants of food in-take. J. Comp Physiol Psychol.

[pcbi-0030097-b007] Le Magnen J, Stellar E, Sprague JM (1971). Advances in studies on the physiological control and regulation of food intake. Progress in physiological psychology, vol. 4.

[pcbi-0030097-b008] Larue-Achagiotis C, Le Magnen J (1982). Effects of short-term nocturnal and diurnal food deprivation on subsequent feeding in intact and VMH lesioned rats: Relation to blood glucose level. Physiol Behav.

[pcbi-0030097-b009] Zorrilla EP, Inoue K, Fekete EM, Tabarin A, Valdez GR (2005). Measuring meals: Structure of prandial food and water intake of rats. Am J Physiol Regul Integr Comp Physiol.

[pcbi-0030097-b010] Gibbs J, Young RC, Smith GP (1973). Cholecystokinin decreases food intake in rats. J Comp Physiol Psychol.

[pcbi-0030097-b011] Ritter S, Dinh T, Friedman M (1994). Induction of Fos-like immunoreactivity (Fos-li) and stimulation of feeding by 2,5-anhydro-D-mannitol (2,5-AM) require the vagus nerve. Brain Res.

[pcbi-0030097-b012] Travers S, Norgren R (1987). Gustatory neural processing in the hindbrain. Annu Rev Neurosci.

[pcbi-0030097-b013] Marx J (2003). Cellular warriors in the battle of the bulge. Science.

[pcbi-0030097-b014] Stein LJ, Woods SC (1982). Gastrin releasing peptide reduces meal size in rats. Peptides.

[pcbi-0030097-b015] Rushing PA, Gibbs J (1998). Prolongation of intermeal interval by gastrin releasing peptide depends upon time of delivery. Peptides.

[pcbi-0030097-b016] Larsen PJ, Fledelius C, Knudsen LB, Tang-Christensen M (2001). Systemic administration of the long-acting GLP-1 derivative NN2211 induces lasting and reversible weight loss in both normal and obese rats. Diabetes.

[pcbi-0030097-b017] Batterham RL, Cowley MA, Small CJ, Herzog H, Cohen MA (2002). Gut hormone PYY(3–36) physiologically inhibits food intake. Nature.

[pcbi-0030097-b018] Chance WT, Balasubramaniam A, Zhang FS, Wimalawansa SJ, Fischer JE (1991). Anorexia following the intrahypothalamic administration of amylin. Brain Res.

[pcbi-0030097-b019] Geary N (1998). Satiation: From gut to brain.

[pcbi-0030097-b020] Woods SC (2004). Gastrointestinal satiety signals I. An overview of gastrointestinal signals that influence food intake. Am J Physiol Gastrointest Liver Physiol.

[pcbi-0030097-b021] Ahren B, Mansson S, Gingerich RL, Havel PJ (1997). Regulation of plasma leptin in mice: Influence of age, high-fat diet and fasting. Am J Physiol Regul Integr Comp Physiol.

[pcbi-0030097-b022] Havel PJ, Kasim Karakas S, Mueller W, Johnson PR, Gingerich RL (1996). Relationship of plasma leptin to plasma insulin and adiposity in normal weight and overweight women: Effects of dietary fat content and sustained weight loss. J Clin Endocrinol Metab.

[pcbi-0030097-b023] Bagdade JD, Bierman EL, Porte D (1967). The significance of basal insulin levels in the evaluation of the insulin response to glucose in diabetic and nondiabetic subjects. J Clin Invest.

[pcbi-0030097-b024] Polonsky KS, Given E, Carter V (1988). Twenty-four-hour profiles and pulsatile patterns of insulin secretion in normal and obese subjects. J Clin Invest.

[pcbi-0030097-b025] Woods SC, Decke E, Vasselli JR (1974). Metabolic hormones and regulation of body weight. Psychol Rev.

[pcbi-0030097-b026] Chelikani PK, Haver AC, Reeve JR, Keire DA, Reidelberger RD (2006). Daily intermittent intravenous infusion of peptide YY(3–36) reduces daily food intake and adiposity in rats. Am J Physiol.

[pcbi-0030097-b027] Batterham RL, Heffron H, Kapoor S, Chivers JE, Chandarana K (2006). Critical role for peptide YY in protein-mediated satiation and body-weight regulation. Cell Metab.

[pcbi-0030097-b028] Roth CL, Enriori PJ, Harz K, Woelfle J, Cowley MA (2005). Peptide YY is a regulator of energy homeostasis in obese children before and after weight loss. J Clin Endocrinol Metab.

[pcbi-0030097-b029] Ellacott KLJ, Halatchev IG, Cone RD (2006). Interactions between gut peptides and the central melanocortin system in the regulation of energy homeostasis. Peptides.

[pcbi-0030097-b030] Smith GP (2000). The controls of eating: A shift from nutritional homeostasis to behavioral neuroscience. Nutrition.

[pcbi-0030097-b031] Bray GA, Fisler J, York DA (1990). Neuroendocrine control of the development of obesity: Understanding gained from studies of experimental animal models. Front Neuroendocrinol.

[pcbi-0030097-b032] Stanley BG, Willett VL, Donias HW, Ha LH, Spears LC (1993). The lateral hypothalamus: A primary site mediating excitatory amino acid–elicited eating. Brain Res.

[pcbi-0030097-b033] Stellar E (1954). The physiology of motivation. Psychol Rev.

[pcbi-0030097-b034] Schwartz MW, Woods SC, Porte DJ, Seeley RJ, Baskin DG (2000). Central nervous system control of food intake. Nature.

[pcbi-0030097-b035] Elmquist J, Maratos-Flier E, Saper C, Flier J (1998). Unraveling the central nervous system pathways underlying responses to leptin. Nature Neurosci.

[pcbi-0030097-b036] Elmquist JK, Flier JS (2004). The fat–brain axis enters a new dimension. Science.

[pcbi-0030097-b037] Cone RD (2005). Anatomy and regulation of the central melanocortin system. Nature Neurosci.

[pcbi-0030097-b038] Fan W, Ellacott KLJ, Halatchev IG, Takahashi K, Yu P (2004). Cholecystokinin-mediated suppression of feeding involves the brainstem melanocortin system. Nature Neurosci.

[pcbi-0030097-b039] Barsh GS, Schwartz MW (2002). Genetic approaches to studying energy balance: Perception and integration. Nat Rev Genet.

[pcbi-0030097-b040] Acuna-Goycolea C, Van Den Pol AN (2005). Peptide YY(3–36) inhibits both anorexigenic proopiomelanocortin and orexigenic neuropeptide Y neurons: Implications for hypothalamic regulation of energy homeostasis. J Neurosci.

[pcbi-0030097-b041] Cowley MA, Smith RG, Diano S, Tschop M, Pronchuk N (2003). The distribution and mechanism of action of ghrelin in the CNS demonstrates a novel hypothalamic circuit regulating energy homeostasis. Neuron.

[pcbi-0030097-b042] Moran TH, Ladenheim EE, Schwartz GJ (2001). Within-meal gut feedback signaling. Int J Obes Relat Metab Disord.

[pcbi-0030097-b043] Schwartz GJ, Moran TH (1996). Sub-diaphragmatic vagal afferent integration of meal-related gastrointestinal signals. Neurosci Biobehav Rev.

[pcbi-0030097-b044] Schwartz GJ, Moran TH, White WO, Ladenheim EE (1997). Relationships between gastric motility and gastric vagal afferent responses to CCK and GRP in rats differ. Am J Physiol Regul Integr Comp Physiol.

[pcbi-0030097-b045] Ahima RS, Saper CB, Flier JS, Elmquist JK (2000). Leptin regulation of neuroendocrine systems. Front Neuroendocrinol.

[pcbi-0030097-b046] Cone RD, Cowley MA, Butler AA, Fan W, Marks DL (2001). The arcuate nucleus as a conduit for diverse signals relevant to energy homeostasis. Int J Obes Relat Metab Disord.

[pcbi-0030097-b047] Niswender KD, Schwartz MW (2003). Insulin and leptin revisited: Adiposity signals with overlapping physiological and intracellular signaling capabilities. Front Neuroendocrinol.

[pcbi-0030097-b048] Obici S, Feng Z, Morgan K, Stein D, Karkanias G (2002). Central administration of oleic acid inhibits glucose production and food intake. Diabetes.

[pcbi-0030097-b049] Wang T, Edwards GL (1997). Differential effects of dorsomedial medulla lesion size on ingestive behavior in rats. Am J Physiol.

[pcbi-0030097-b050] Grill HJ, Kaplan JM (2002). The neuroanatomical axis for control of energy balance. Front Neuroendocrinol.

[pcbi-0030097-b051] Staddon JER, Zanutto BS, Bouton ME, Fanselow MS (1997). Feeding dynamics: Why rats eat in meals and what this means for foraging and feeding regulation. Learning, motivation and cognition: The functional behaviorism of Robert C. Bolles.

[pcbi-0030097-b052] Staddon JER (2001). Adaptive dynamics: The theoretical analysis of behavior.

[pcbi-0030097-b053] Levitsky D (2000). Putting behavior into feeding behavior: A tribute to George Collier. Appetite.

[pcbi-0030097-b054] Strubbe JH, Woods SC (2004). The timing of meals. Psychol Rev.

[pcbi-0030097-b055] Le Magnen J (1985). Hunger.

