# Correction: Bang-Bang Control of Feeding: Role of Hypothalamic and Satiety Signals

**DOI:** 10.1371/journal.pcbi.0030127

**Published:** 2007-06-29

**Authors:** B. Silvano Zanutto, John E. R Staddon

In *PLoS Computational Biology*, volume 3, issue 5, in the Introduction section: doi:10.1371/journal.pcbi.0030097

The word glucagon was misspelled glucagons. This is the correct sentence:

In addition, amylin [18] and glucagon [19], which are secreted from the pancreatic islets during meals, also reduce meal size.

The following sentence was missing a reference (34):

Conversely, bilateral PVN lesions cause a hyperphagic obesity syndrome, whereas bilateral lesioning of the LHA causes anorexia and weight loss [31,33,34].

The following sentence had an incorrect reference (52 is correct instead of 50):

The bang-bang CINT model also accounts quantitatively for the complexities of meal-intermeal correlations [52] (see also [9,54]).

